# Control of tissue homeostasis, tumorigenesis, and degeneration by coupled bidirectional bistable switches

**DOI:** 10.1371/journal.pcbi.1009606

**Published:** 2021-11-19

**Authors:** Diego Barra Avila, Juan R. Melendez-Alvarez, Xiao-Jun Tian

**Affiliations:** School of Biological and Health Systems Engineering, Arizona State University, Tempe, Arizona, United States of America; Duke University, UNITED STATES

## Abstract

The Hippo-YAP/TAZ signaling pathway plays a critical role in tissue homeostasis, tumorigenesis, and degeneration disorders. The regulation of YAP/TAZ levels is controlled by a complex regulatory network, where several feedback loops have been identified. However, it remains elusive how these feedback loops contain the YAP/TAZ levels and maintain the system in a healthy physiological state or trap the system in pathological conditions. Here, a mathematical model was developed to represent the YAP/TAZ regulatory network. Through theoretical analyses, three distinct states that designate the one physiological and two pathological outcomes were found. The transition from the physiological state to the two pathological states is mechanistically controlled by coupled bidirectional bistable switches, which are robust to parametric variation and stochastic fluctuations at the molecular level. This work provides a mechanistic understanding of the regulation and dysregulation of YAP/TAZ levels in tissue state transitions.

## Author summary

Tissue development and homeostasis require well-controlled cell proliferation. Lack of this control could lead to degenerative or tumorigenic diseases. Signaling pathways have been explored in promoting or inhibiting these diseases. The Hippo signaling pathway is one of these, which has been found to control tissue homeostasis and organ size through cell proliferation and apoptosis, as evidenced by extensive experimental data. However, the question remains of how tissue can transition from a homeostatic state to either a degenerative or tumorigenic state. By theoretically analyzing a mathematical model of its regulatory network, we present a mechanism that underlies Hippo signaling to control tissue transition from a homeostatic state to a disease state. This provides us with a mechanistic understanding of how the parts of the regulatory network are coordinated for the transitions between the homeostasis state and the disease states. In addition, we looked at the role of system noise and found that it could promote the transition to one of the disease states. Our model allows for experimental hypotheses to be generated and could lead to the development of therapeutic strategies by targeting the Hippo signaling pathway.

## Introduction

The Hippo signaling pathway is responsible for organ size control, tissue homeostasis, and regeneration [1–3]. Dysfunction of this pathway has been associated with tumorigenesis and degenerative diseases [1, 4–6]. This pathway consists of several kinases that target two transcriptional coactivators, Yes-associated protein 1 (YAP) and PDZ-binding motif (TAZ) [1–6]. Following the activation of the Hippo pathway, a phosphorylation cascade starting with NF2 leads to sequential activation of MST1/2 and LATS1/2, which further phosphorylates YAP/TAZ at specific serine residues, leading to the sequestration of YAP/TAZ in the cytoplasm for degradation via the ubiquitin-proteasome pathway [1–6]. When Hippo is inactivated, YAP/TAZ remains unphosphorylated and enters the nucleus, where it binds to several cofactors, such as TEAD, RUNX, or SMAD, and regulates gene expression of many targets [1–6]. Some of these targets also regulate the levels of YAP/TAZ, and thus a complex regulatory network with many feedback loops is formed. However, the roles of these feedbacks loops remain underexplored.

Homeostasis was proposed to be maintained through a negative feedback loop between YAP/TAZ and LATS1/2, where YAP/TAZ promotes LATS1/2 expression through TEAD/NF2 while LATS1/2 inactivates YAP/TAZ [7]. Dysfunction of Hippo signaling could lead to unregulated YAP/TAZ activity and thus increased cell proliferation, leading to tumorigenesis [1, 4]. But lack of YAP/TAZ activity and expression of its target genes has been observed to cause developmental defects and tissue degeneration [1, 5, 6]. Thus, proper regulation of the Hippo signaling pathway allows for tissue to exist in a homeostatic state, but improper regulation can trap tissue in diseased states, either degenerative or tumorigenic.

Crosstalk between the Hippo-YAP/TAZ and Notch signaling pathway has been observed in several cancers. Notch ligand Jag1 was dependent on YAP/TAZ activity, and Notch intracellular domain (NICD) reduced TAZ degradation, thus placing YAP/TAZ and Notch signaling in a positive feedback loop in hepatocellular carcinoma (HCC) [8]. More evidence of this positive feedback loop can be found in rhabdomyosarcomas, as the core Notch transcription RPBJ regulated YAP1 through direct transcription, and NICD overexpression increased YAP1 levels [9]. Another positive feedback loop between YAP/TAZ and SIRT1 is associated with different types of tissue degeneration, such as neurodegeneration and retinal degeneration [1, 5, 6, 10, 11]. SIRT1 can promote YAP through Pol II-dependent transcription [12], even promote its activity through deacetylation [10], while YAP can promote SIRT1 expression through Myc [10, 12, 13]. Thus, the YAP/TAZ level is regulated by several coupled feedback loops. However, it remains elusive how these feedback loops are orchestrated to control the YAP/TAZ level and thus the transition from tissue homeostasis to tumorigenesis or degenerative diseases.

In this work, we developed a mathematical model to recapitulate YAP/TAZ regulatory network. Through theoretical analyses, we found three states with distinct levels of YAP/TAZ that designate the tissue homeostasis, tumorigenesis, and degeneration states, respectively. Coupled bidirectional bistable switches (CBBS) were found to control the transition from the homeostatic state to the tumorigenic or degenerative states. The homeostatic state is controlled by the YAP/TAZ/LATS negative feedback loop, and the bidirectional bistable switches are governed by coupled positive feedback loops. In addition, these conclusions are robust to parametric variation and molecular-level fluctuations.

## Results

### Mathematical model for the Hippo-YAP/TAZ signaling pathway

**Fig 1:**
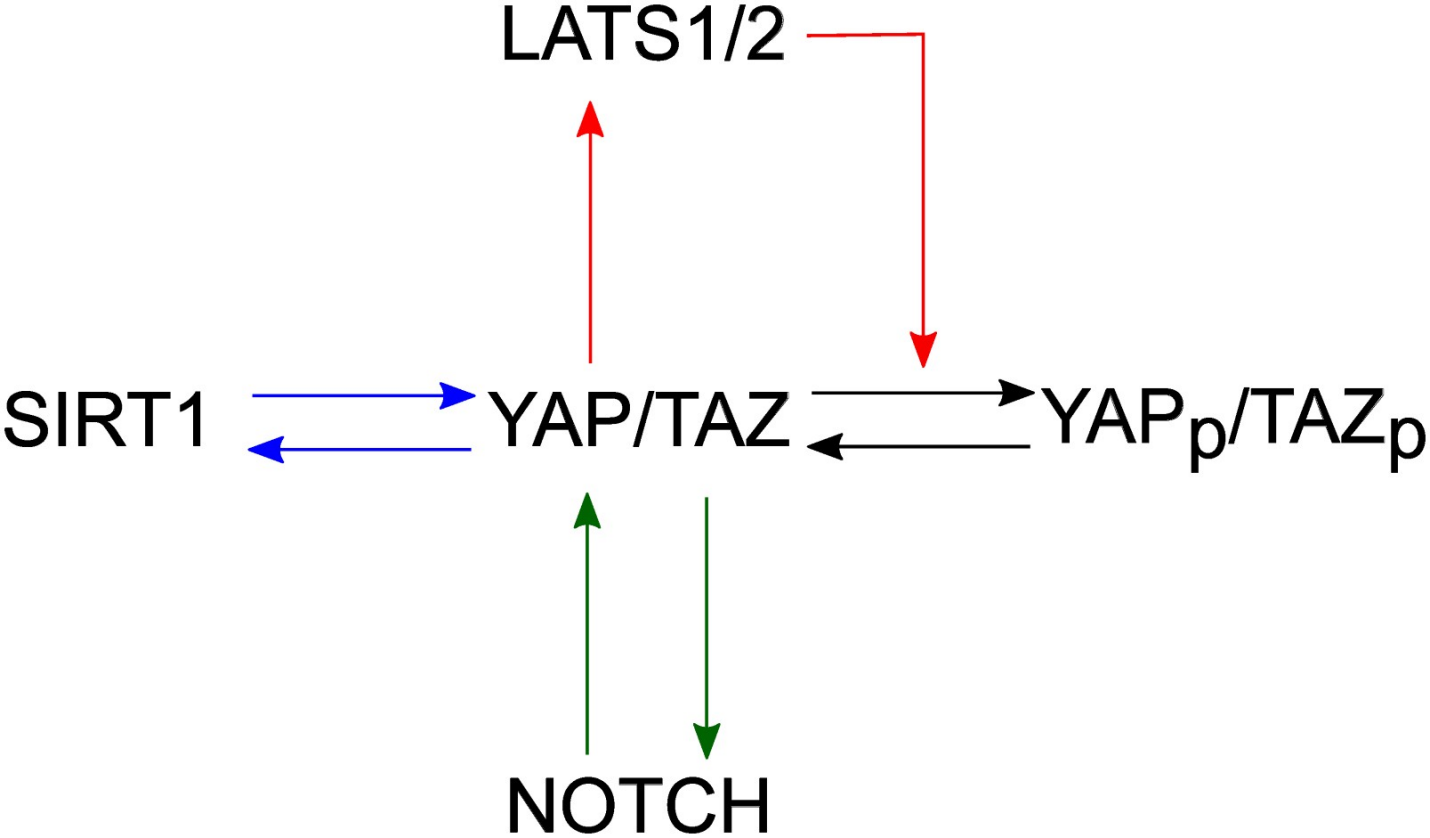
Schematic depiction of the regulatory network of YAP/TAZ. LATS1/2 promotes the phosphorylation of YAP/TAZ [1–6], leading cytoplasmic accumulation and subsequent degradation of YAP/TAZ while YAP/TAZ promotes the transcription of LATS2 [7]. YAP/TAZ promotes the Notch signaling pathway by inducing transcription of JAG1, DLL1, and RBPJ [8, 9], while Notch signaling pathway promotes YAP/TAZ expression through RBPJ-binding to YAP1 promoter [8, 9]. SIRT1 promotes YAP through Pol II-dependent transcription [12], while YAP/TAZ promotes SIRT1 transcription by upregulating MYC [13, 14].

To understand the regulation of YAP/TAZ activity and levels and its roles in tissue homeostasis, tumorigenesis, and degeneration, we built a mathematical model based on the regulatory network in Fig 1. The Hippo signaling pathway regulates YAP/TAZ activity through phosphorylation. Upon Hippo signaling activation, LATS1/2 is activated to phosphorylate YAP/TAZ [1–6]. Dephosphorylation of YAP/TAZ is regulated by GPCR signaling [7]. In unphosphorylated form, YAP/TAZ stays in the nucleus and induces transcription of LATS2 [1], forming a negative feedback loop (Fig 1, red lines). The interactions between unphosphorylated YAP/TAZ and SIRT1 form one positive feedback loop (Fig 1, blue lines), in which SIRT1 promotes the transcription of YAP, while YAP promotes transcription of SIRT1 [10, 12, 13]. YAP/TAZ and Notch form another positive feedback loop (Fig 1, green line), in which YAP/TAZ activates Notch signaling by transcribing for Notch ligands and Notch promotes transcription of YAP [8, 9]. We used the following ordinary differential equations (ODEs) to describe the deterministic behavior of this regulatory network.

$$\begin{array}{ccc} \frac{d[L]}{dt} & = & k_{L1}+k_{L2}\cdot \frac{{\left[YT_{up}\right]}^{n}}{{\left[YT_{up}\right]}^{n}+J_{L}^{n}}-k_{L3}\cdot \left[L\right], \end{array}$$ (1)

$$\begin{array}{ccc} \frac{d[\;YT_{up}]\;}{dt} & = & k_{YTup0}+k_{YTup1}\cdot \frac{{\left[S\right]}^{n}}{{\left[S\right]}^{n}+J_{YTup1}^{n}}\\ & & +k_{YTup2}\cdot \frac{{\left[N\right]}^{n}}{{\left[N\right]}^{n}+J_{YTup2}^{n}}-k_{YTup3}\cdot \frac{\left[YT_{up}\right]\cdot \left[L\right]}{\left[YT_{up}\right]+J_{YTup3}}\\ & & +k_{YTup4}\cdot \frac{\left[YT_{p}\right]}{\left[YT_{p}\right]+J_{YTup4}}-k_{YTup5}\cdot \left[YT_{up}\right], \end{array}$$ (2)

$$\begin{array}{ccc} \frac{d[YT_{p}]}{dt} & = & k_{YTup3}\cdot \frac{\left[YT_{up}\right]\cdot \left[L\right]}{\left[YT_{up}\right]+J_{YTup3}}-k_{YTup4}\cdot \frac{\left[YT_{p}\right]}{\left[YT_{p}\right]+J_{YTup4}}\\ & & -k_{YTp1}\cdot \left[YTp\right], \end{array}$$ (3)

$$\begin{array}{ccc} \frac{d[S]}{dt} & = & k_{S1}+k_{S2}\cdot \frac{{\left[YT_{up}\right]}^{n}}{{\left[YT_{up}\right]}^{n}+J_{S}^{n}}-k_{S3}\cdot \left[S\right], \end{array}$$ (4)

$$\begin{array}{ccc} \frac{d[N]}{dt} & = & k_{N1}+k_{N2}\cdot \frac{{\left[YT_{up}\right]}^{n}}{{\left[YT_{up}\right]}^{n}+J_{N}^{n}}-k_{N3}\cdot \left[N\right], \end{array}$$ (5)

in which [*L*], [*YT*_*up*_], [*YT*_*p*_], [*S*], and [*N*] denote the concentrations of endogenous LATS1/2, unphosphorylated YAP/TAZ, phosphorylated YAP/TAZ, SIRT1, and NOTCH, respectively. For the phosphorylation and dephosphorylation reactions of YAP/TAZ, Michaelis-Menten kinetics were used. The function $\frac{\left[YT_{up}\right]\cdot \left[L\right]}{\left[YT_{up}\right]+J_{YTup3}}$ represents the phosphorylation of YAP/TAZ by LATS1/2. The function $\frac{\left[YT_{p}\right]}{\left[YT_{p}\right]+J_{YTup4}}$ was used to represent the dephosphorylation of YAP/TAZ. Hill functions $\frac{J_{X_{i}}^{n}}{{\left[X_{i}\right]}^{n}+J_{X_{i}}^{n}}$ and $\frac{{\left[X_{i}\right]}^{n}}{{\left[X_{i}\right]}^{n}+J_{X_{i}}^{n}}$ were used for all the transcription regulation in the network. In addition, the basal production rate was assumed as constant, and the degradation rate was assumed proportional to the protein concentration [15–17], with the degradation rate constant estimated using the half-life of each protein [18, 19]. Table A in S1 Text summarizes the description of differential equation terms. Table B and C in S1 Text list the definition and the initial value of each variable and a set of standard parameter values, respectively. The default state is the tissue homeostatic state, in which the concentration of each species is set to its stable steady-state value under the standard parameter set.

In addition, our 5-ODE system can be reduced to a 2-ODE system for further analysis:

$$\begin{array}{ccc} \frac{d[L]}{dt} & = & k_{L1}+k_{L2}\cdot \frac{{\left[YT_{up}\right]}^{n}}{{\left[YT_{up}\right]}^{n}+J_{L}^{n}}-k_{L3}\cdot \left[L\right], \end{array}$$ (6)

$$\begin{array}{ccc} \frac{d[\;YT_{up}]\;}{dt} & = & k_{YTup0}+k_{YTup1}\cdot \frac{{\left[S^{*}\right]}^{n}}{{\left[S^{*}\right]}^{n}+J_{YTup1}^{n}}\\ & & +k_{YTup2}\cdot \frac{{\left[N^{*}\right]}^{n}}{{\left[N^{*}\right]}^{n}+J_{YTup2}^{n}}-k_{YTup3}\cdot \frac{\left[YT_{up}\right]\cdot \left[L\right]}{\left[YT_{up}\right]+J_{YTup3}}\\ & & +k_{YTup4}\cdot \frac{\left[YT_{p}^{*}\right]}{\left[YT_{p}^{*}\right]+J_{YTup4}}-k_{YTup5}\cdot \left[YT_{up}\right], \end{array}$$ (7)

where [S*],[N*], and $\left[YT_{p}^{*}\right]$ are the steady-state concentrations that follows

$$\begin{array}{ccc} \left[S^{*}\right] & = & \frac{1}{k_{S3}}\cdot (k_{S1}+k_{S2}\cdot \frac{{\left[YT_{up}\right]}^{n}}{{\left[YT_{up}\right]}^{n}+J_{S}^{n}}), \end{array}$$ (8)

$$\begin{array}{ccc} \left[N^{*}\right] & = & \frac{1}{k_{N3}}\cdot (k_{N1}+k_{N2}\cdot \frac{{\left[YT_{up}\right]}^{n}}{{\left[YT_{up}\right]}^{n}+J_{N}^{n}}), \end{array}$$ (9)

$$\begin{array}{ccc} \left[YT_{p}^{*}\right] & = & \frac{1}{2\cdot k_{YTp1}}[(k_{YTup3}\cdot \frac{\left[YT_{up}\right]\cdot \left[L\right]}{\left[YT_{up}\right]+J_{YTup3}}-k_{YTup4}\\ & & -k_{YTp1}\cdot J_{YTup4})+((-k_{YTup3}\cdot \frac{\left[YT_{up}\right]\cdot \left[L\right]}{\left[YT_{up}\right]+J_{YTup3}}\\ & & +k_{YTup4}+k_{YTp1}\cdot J_{YTup4}{)}^{2}\\ & & +4\cdot k_{YTp1}\cdot k_{YTup3}\cdot \frac{\left[YT_{up}\right]\cdot \left[L\right]}{\left[YT_{up}\right]+J_{YTup3}}\cdot J_{YTup4}{)}^{0.5}]. \end{array}$$ (10)

### The transition to tumorigenesis or degeneration disease is regulated by two coupled bi-directional bistable switches

**Fig 2:**
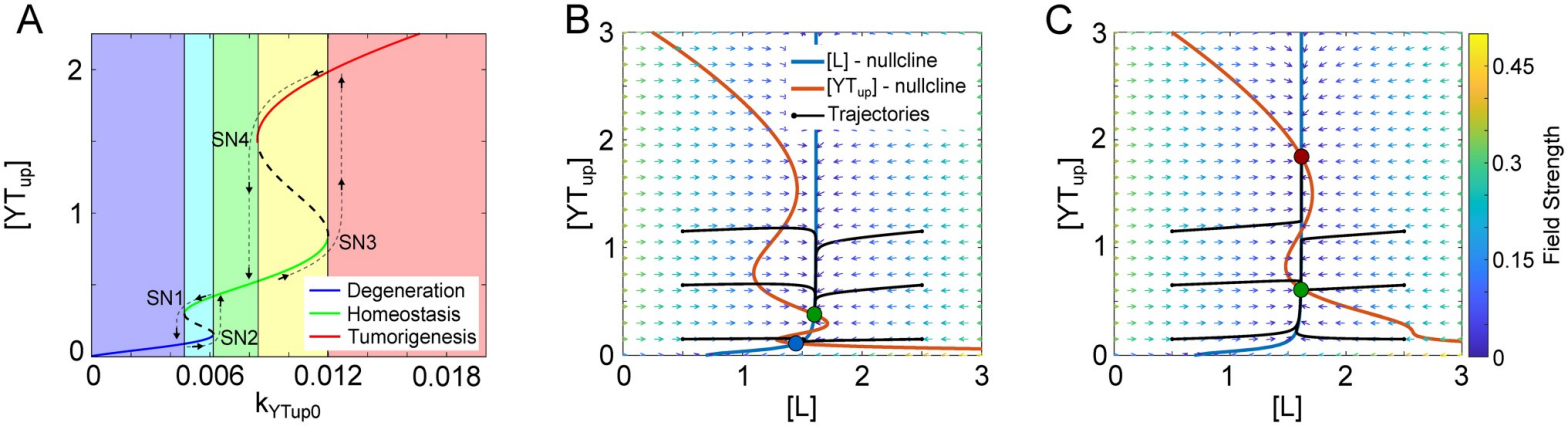
Bifurcation and nullcline analysis displays three states. (A) The bifurcation diagram shows the steady state of active/unphosphorylated YAP/TAZ as a function of basal expression of unphosphorylated YAP/TAZ (*k*_*YTup*0_). The labeled points SN1, SN2, SN3, and SN4 indicate saddle-node bifurcation points. The black dashed lines represent the unstable steady states while the solid colored lines represent the steady states. The lower blue branch, middle green branch, and upper red branch are defined as the degenerative state, homeostatic state, and tumorigenic state, respectively. The green shaded region shows the monostable range within the homeostatic state, the blue shaded region shows monostable range within the degenerative state, and the red shaded region shows the monostable range within the tumorigenic state. The cyan shaded region shows a bistable region with degenerative and homeostatic states, while the yellow shaded region shows the bistable region with the homeostatic and tumorigenic states. (B) Nullcline analysis of the regulatory system shows the two stable steady states and one unstable steady state when *k*_*YTup*0_ is within the first bistable region (between SN1 and SN2). Example trajectories starting from different initial conditions were shown to represent the dynamics of the system towards the tissue homeostatic and degenerative states. (C) Nullcline analysis of the regulatory system shows the two stable steady states and one unstable steady state when *k*_*YTup*0_ is within the second bistable region (between SN3 and SN4). Example trajectories (black solid lines) starting from different initial conditions were shown to represent the dynamics of the system towards the tissue homeostatic and tumorigenic states.

To understand how the YAP/TAZ levels are controlled by the regulatory network and how tissue states are determined by YAP/TAZ dynamics, we performed a one-parameter bifurcation analysis over the basal production rate of YAP/TAZ (*k*_*YTup*0_). Here we used *k*_*YTup*0_ as the bifurcation parameter to represent any mutation or dysregulation in the system that could change the production rate of YAP/TAZ and thus change the tissue state. As shown in Fig 2A, the dependence of the steady states of unphosphorylated YAP/TAZ on *k*_*YTup*0_ shows three different types of stable steady states. The middle branch (green line) with moderate levels of YAP/TAZ corresponds to the tissue homeostasis state, while the bottom branch (blue line) with low levels of YAP/TAZ and the upper branch (red lines) with high levels of YAP/TAZ correspond to the degenerative and tumorigenic tissue states, respectively. This is consistent with the findings that reduced levels of YAP/TAZ were found in degenerative diseases and increased levels of YAP/TAZ were found in tumorigenic models [1, 5–9, 13, 20, 21]. Furthermore, degeneration diseases were found to have lower levels of SIRT1 and NOTCH [22–25], while tumorigenic diseases had higher levels of SIRT1 and NOTCH [8–10, 12, 14].

The bifurcation diagram shows that two coupled bi-directional bistable switches are responsible for transitions among these tissue states. It is noted that the system is monostable in the homeostatic state with a small variation of *k*_*YTup*0_ (green shaded region). However, as the value of *k*_*YTup*0_ reaches the saddle-node bifurcation point (SN1), the system transitions from the homeostatic state to the degenerative state (Fig 2A, dashed lines with black arrows), which is controlled by the first bistable switch. The system can be recovered back only if *k*_*YTup*0_ reaches another threshold SN2, which is higher than SN1. That is, the system is monostable and immersed in the degenerative state when *k*_*YTup*0_ < *SN*1 (blue shaded region) but shows bistability when *SN*1 < *k*_*YTup*0_ < *SN*2 (cyan shaded region). The bistability in this region is also observed in the nullcline analysis (Fig 2B). It is noted that [L]-nullcline is unaffected by the *k*_*YTup*0_ value, as it is independent of this parameter according to the 2-ODE system. The [*YT*_*up*_]-nullcline shifts to the left as *k*_*YTup*0_ decreases, and thus intersect with [L]-nullcline at three steady states, two of which are stable steady states, corresponding to the homeostatic state (Fig 2B, green circle) and the degenerative state (Fig 2B, blue circle).

The second switch triggers the transition from the homeostatic state to the tumorigenic state (Fig 2A, dashed lines with black arrows), as *k*_*YTup*0_ reaches the saddle-node bifurcation point (SN3). The system can be recovered back only if *k*_*YTup*0_ reaches another threshold SN4, which is lower than SN3. The system is trapped in the tumorigenic state when *k*_*YTup*0_ > *SN*3 (red shaded region) and shows bistability when *SN*4 < *k*_*YTup*0_ < *SN*3 (yellow shaded region). In the phase plane (Fig 2C), the [*YT*_*up*_]-nullcline shifts to the right as a result of increased *k*_*YTup*0_ and thus intersects with the L-nullcline with three steady steady states, two of which are stable and correspond to the homeostatic state (Fig 2C, green circle) and the tumorigenic state (Fig 2C, red circle). That is, this bistable switch can ramp down to the degeneration state or switch up to the tumorigenesis state, showing a bidirectional nature. Thus, the transition from the tissue homeostasis state to tumorigenesis or degeneration disease is regulated by two coupled bi-directional bistable switches.

### Tissue homeostasis is regulated by YAP/TAZ/LATS negative feedback loop

**Fig 3:**
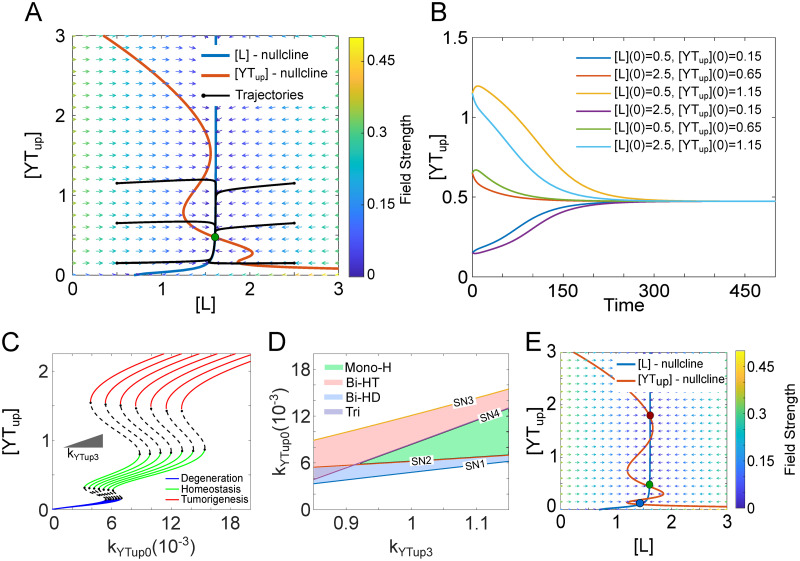
Tissue homeostasis is regulated by YAP/TAZ/LATS negative feedback loop. (A) Nullcline analysis of the regulatory system shows one stable steady state (tissue homeostasis, green circle) at the intersections of the nullclines under basal parameter set. The [*L*]-nullcline is drawn in blue and the [*YT*_*up*_]-nullcline is drawn in red. The vector field of the system is represented by small arrows, where the color is proportional to the field strength. Example trajectories starting from different initial states were shown to represent the dynamics of the system towards the tissue homeostatic state. (B) The time course of the [*YT*_*up*_] level with the system starting from a range of initial conditions. (C) The parameter *k*_*YTup*3_, characterizing the strength of the YAP/TAZ-LATS1/2 negative feedback loop, was varied from 85% to 115% (left to right), with 5% increments, of its original value to plot the 1-parameter bifurcation diagrams and show its effect on the homeostatic state. (D) Two-parameter bifurcation analysis shows how the thresholds SN1, SN2, SN3, and SN4 vary with *k*_*YTup*3_. The monostable homeostatic state region is labeled as Mono-H (green), and the tristable region is labeled as Tri (purple), respectively. The bistable region in which the homeostatic and tumorigenic states existed within is labeled as Bi-HT (red), while the bistable region in which the homeostatic and degenerative states existed within is labeled as Bi-HD (blue). (E) Nullcline analysis of the regulatory system shows the three stable steady states and two unstable steady states when *k*_*YTup*0_ is within the tristable region (between SN2 and SN4) and *k*_*YTup*3_ is 85% of its original value.

The Hippo signaling pathway regulates tissue homeostasis by using the YAP/TAZ-LATS1/2 negative feedback loop. We performed nullcline analysis to study how this negative feedback loop maintains tissue homeostasis. As shown in Fig 3A, the system only has one stable steady state at the intersection of the [*YT*_*up*_] and [*L*] nullclines under the standard parameter set. This steady state corresponds to the homeostasis state. The time course of [*YT*_*up*_] using the reduced 2-ODE model shows that for all various initial conditions, the system converges to the stable steady state. The vector field and trajectories in Fig 3A show that the [*L*] steady state is reached before the [*YT*_*up*_] steady state is. For different initial conditions, Fig 3B shows YAP/TAZ reaching its steady state after some time has passed. This analysis allows us to see how the negative feedback loop between YAP/TAZ and LATS1/2 operates to maintain tissue homeostasis.

To further understand the importance of the YAP/TAZ-LATS1/2 negative feedback loop in maintaining homeostasis, we explored the effect of its strength on the range of monostable homeostasis state within the 1-parameter bifurcation diagram (Fig 2A). We perturbed the strength of this negative feedback by varying one parameter *k*_*YTup*3_, the phosphorylation rate of YAP/TAZ by LATS1/2, from 85% to 115% of its original value, and studied how the bifurcation diagram changes. We found that by increasing the negative feedback strength *k*_*YTup*3_, the range of homeostatic state increases (Fig 3C). This range is determined by the threshold SN1 and SN3. The dependence of SN1 and SN3 on the negative feedback strength *k*_*YTup*3_ is shown in the two-parameter diagram (Fig 3D). We can see that both SN1 and SN3 increase with *k*_*YTup*3_ but SN3 increases faster, and thus leading to the increased range of homeostatic state.

It is noted that the increase of SN3 with *k*_*YTup*3_ makes the transition from homeostatic state to the tumorigenic state difficult, but SN1 is increased as well at the time, which makes the transition from the homeostatic state to the degenerative state easier. Thus, the system faces a fundamental trade-off between these two pathological transitions; reducing the risk of tumorigenesis increases the risk of degeneration and vice versa. This result also suggests that targeting the YAP/TAZ-LATS1/2 negative feedback needs a good balance to reduce the risks of both degeneration diseases and tumorigenesis.

It is worth noting that when the negative feedback strength is small, the threshold SN4 could be smaller than SN2, which allows the coexistence of all three tissue states under the same condition. That is, the system shows tristability, and the monostable range of the homeostatic state vanished. In the two-parameter bifurcation diagram, the SN4 curve intersects with the SN2 curve at one point, which shows the transition from tristability to a monostable homeostatic state (Fig 3D). Fig 3E shows the nullcline analysis when the system exhibits tristability, which is not good because the system may jump from the homeostasis state to either tumorigenesis or degeneration state through some transient signals. Thus, there is an optimal strength of the YAP/TAZ-LATS1/2 negative feedback loop to make the monostable range of homeostasis state large and both the risks of both the transitions to tumorigenesis and degeneration small.

### Bi-directional bistable switches is orchestrated by coupled positive feedback loops

**Fig 4:**
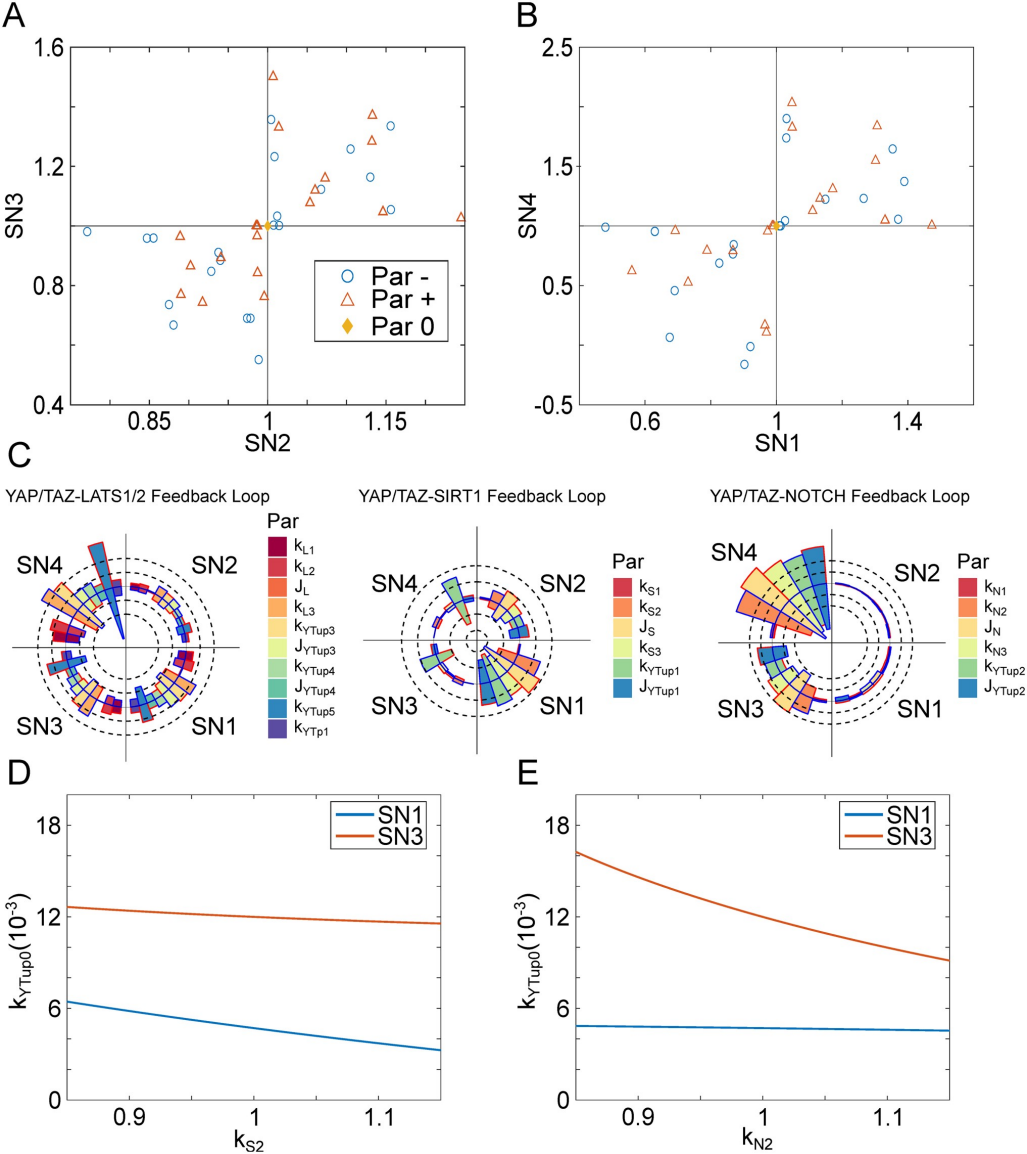
The coupled bidirectional bistable switches are robust to parameter variation. (A-B) Parameter sensitivity analysis was performed for the transition thresholds SN2 and SN3 (A) and SN1 and SN4 (B) by individually increasing and decreasing each parameter by 15% and plotting the fold change. Par- and Par+ represent the increase or decrease of parameters, respectively. Par0 represents the set of standard parameter. The diamond shaped-marker shows the thresholds under the standard parameter set. (C) The circular bar plots show the percent changes in the thresholds when each of the parameters is increased or decreased by 15%. The red outline on the bars denotes an increase of 15% in the parameters, while the blue outline denotes a decrease of 15% in the parameters. The solid black circle represents a 0% change. The dotted black circles going outward and inward represent an increase and decrease of 25% change, respectively. (D-E) Two-parameter bifurcation analyses show the dependence of normalized thresholds for activation of tissue degeneration and tumorigenesis on the strength of (D) the YAP/TAZ-SIRT1 feedback loop (*k*_*S*2_), and (E) YAP/TAZ-NOTCH feedback loop (*k*_*N*2_). Par: Parameter.

To understand the design principle of the bi-directional bistable switches, we performed a parameter sensitivity analysis for the transition thresholds (bifurcation points SN1–4) by increasing and decreasing the value of each parameter by 15%. The four bifurcations points were robustly found to these parameter variations, meaning the saddle nodes continued to exist even with the 15% parameter variation. That is, all three states exist with these parameter variations. The fold changes of these thresholds were analyzed with each parameter variation were shown in Fig 4A and 4B. Thus, the bi-directional bistable switches display as a mechanism that is robust to this parameter variation. We also find some parameter variation could make the tumorigenic state irreversible. For example, in the bifurcation diagram with a decrease of 15% in the parameter *J*_*N*_ (Fig A in S1 Text), SN4 becomes negative, so once the system moves past SN3 into the tumorigenic state, it will not be possible to transition back into any of the other two states.

In addition, we determined which parameters both switches are more sensitive to. We found that the thresholds for the first switch (SN1 and SN2) are more sensitive to the parameters in YAP/TAZ-SIRT1 positive feedback loop, while the thresholds for the second switch (SN3 and SN4) are more sensitive to the parameters in the YAP/TAZ-NOTCH positive feedback loop (Fig 4C). We found the thresholds for both switches to be sensitive to the parameters in the YAP/TAZ-LATS1/2 negative feedback loop, which shows the importance of this negative feedback loop in the regulation of all three states. This is consistent with Fig 3C and 3D, where the YAP/TAZ-LATS1/2 negative feedback loop strength was found to have a significant impact in increasing or decreasing the risk of each of the disease states.

To further demonstrate the dependence of thresholds for degeneration and tumorigenesis on the YAP/TAZ-SIRT1 and YAP/TAZ-NOTCH positive feedback loops, we performed a 2-parameter bifurcation analysis. Fig 4D and 4E show the dependence of these thresholds on the feedback strengths of the two loops, the YAP/TAZ-mediated production rate of SIRT1 (*k*_*S*2_) for the YAP/TAZ-SIRT1 loop, while the YAP/TAZ-mediated production rate of NOTCH (*k*_*N*2_) for the second loop. A change in *k*_*S*2_ or *k*_*N*2_ could move the positions of the saddle nodes and branches that define the states within the 1-parameter bifurcation diagram (Fig 2A). To determine how sensitive the transition from homeostatic state to the two disease states is to *k*_*S*2_ or *k*_*N*2_, we varied them by 15%. Fig 4D and 4E display the dependence of the thresholds for degeneration and tumorigenesis, in terms of *k*_*YTup*0_, on these two parameters. Smaller changes in the y-axis of Fig 4D and 4E can be considered as the saddle nodes being less sensitive to parameter variation. We found that SN1 is sensitive to the *k*_*S*2_, suggesting the induction of degeneration is more regulated by the YAP/TAZ-SIRT1 feedback loop. In comparison, the threshold for tumorigenesis (SN3) is sensitive to *k*_*N*2_, suggesting the regulation of tumorigenesis by the YAP/TAZ-NOTCH feedback loop.

### Influences of noise on the tissue state transitions

Extrinsic and intrinsic noises could affect the transitions between the defined states. Extrinsic noise can occur due to model parameter variations from cell-cell variability and fluctuations in the tissue microenvironment, while intrinsic noise is caused by the stochastic biochemical reactions from low numbers of molecules per cell. To model these types of noise, here we used a Tau-leap-based Gillespie algorithm to include intrinsic noise [26] and/or varied the parameters within the system by 5% to account for the extrinsic noise to study the cell phenotypic heterogeneity at the population level.

**Fig 5:**
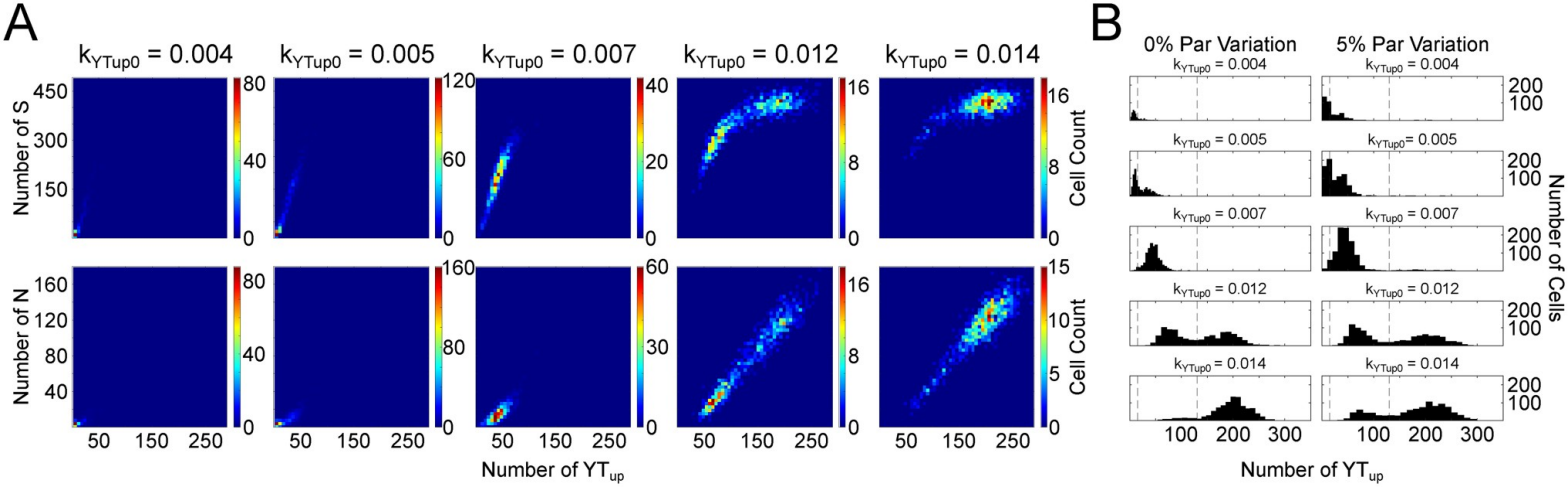
Effect of intrinsic and extrinsic noise on tissue state transitions. (A) The steady 2-D distribution of *N*, *S*, and *YT*_*up*_ from 1000 samples of stochastic simulations by increasing or decreasing *k*_*YTup*0_ value from the standard value *k*_*YTup*0_ = 0.007 that corresponds to the homeostatic state. (B) The steady 1-D distributions of *YT*_*up*_ from 1000 samples of stochastic simulations with only intrinsic noises (left), or both extrinsic and intrinsic noises (right).

We first converted our ODE model to a stochastic model by considering intrinsic noise into the system (Table D in S1 Text). We analyzed the steady distribution of *N*, *S*, and *YT*_*up*_ with 1000 samples of stochastic simulations by increasing or decreasing *k*_*YTup*0_ value from the standard value that corresponds to the homeostatic state. As shown in Fig 5A, most cells stay in the homeostatic state with moderate levels of *N*, *S*, and *YT*_*up*_ under the standard value of *k*_*YTup*0_ = 0.007 (middle panel). We observed that more and more cells transition to the degenerative state with reduced levels of *N*, *S*, and *YT*_*up*_ when *k*_*YTup*0_ value was reduced to 0.005 and 0.004 (Fig 5A, left two panels), indicating the increased risk of degeneration. While more and more cells transition to the tumorigenic state with high levels of *N*, *S*, and *YT*_*up*_ when *k*_*YTup*0_ value was increased set to 0.012 and 0.014 (Fig 5A, right two panels), indicating the increase of risk of tumorigenesis. The transitions between the states can be observed in Fig B of S1 Text, where we display the system dynamics with randomly-picked 10 stochastic simulations. We then added extrinsic noise to the system by setting a varied parameter set to each cell. As shown in Fig 5B, the steady distribution of *YT*_*up*_ with 1000 samples shows more heterogeneities in the system with both noises (right) when compared with the system with only intrinsic noise (left).

## Discussion

Tissue homeostasis requires a balance between cell proliferation and apoptosis. Disruption of this balance allows for the system to transition from the physiological state to the pathological ones. Examining the role of Hippo signaling would provide an insight into how these transitions occur. The analysis performed on the regulatory network representing the Hippo signaling pathway showed how it regulates homeostasis, the transitions between physiological and pathological states. Regulatory networks with multiple positive feedback loops have been observed to regulate other cell phenotype transitions through coupled bistable switches, such as epithelial-to-mesenchymal transition (EMT) [18, 27–30]. Same as the cascading bistable switches (CBS) [18, 27] and Ternary Chimera Switch (TCS) mechanisms [28], here two coupled positive feedback loops account for the multistability. However, different from those mechanisms, we found that the Hippo pathway regulates the transition between physiological and pathological states with the coupled bidirectional bistable switches. Specifically, the base state (homeostasis) can switch up to the tumorigenic state or switch down to the degenerative condition. Negative feedback loops are found in numerous biological systems to maintain homeostasis and robustness to perturbations [31, 32]. Here, the YAP/TAZ-LATS1/2 negative feedback loop strength was observed to have an impact on both the range of homeostasis as well as the range of monostability within this homeostatic region. Furthermore, we found that LATS1/2 first had to reach its steady state before repressing YAP/TAZ, indicating a time delay such that YAP/TAZ has enough time to act as transcriptional coactivators [7]. This mechanism displays the importance of the additional YAP/TAZ-LATS1/2 negative feedback in the system towards maintaining the tissue homeostasis state. We also found a fundamental tradeoff between reducing the risk of the system transitioning to the two pathological transitions by tuning the YAP/TAZ-LATS1/2 negative feedback loop, which is from the double-edged nature of the system and poses a dilemma for treatment with this negative feedback loop as the therapeutic target.

Though it would be more beneficial for cells to have only the homeostatic state, cells have other functions and require high and low levels of YAP/TAZ. For example, higher levels of YAP/TAZ are needed for tissue regeneration of the liver, heart, and skin [3]. In the case of having lower levels of YAP/TAZ, as organisms grow, they might not require as much YAP/TAZ as they did during early development. At later life stages, the level of YAP/TAZ could be slowed down in cells of a particular tissue. One possibility is that tissues might only have a certain percentage of cells changing their levels of YAP/TAZ to achieve these other functions. It is also possible that random mutations can change the expression levels of YAP/TAZ, thus triggering the system into one of the disease states. In cases where the percentage of these cells that commit to one disease state increases, the risk of the tissue transitioning to this state also increases.

Our analysis to determine the effect of extrinsic noise on tissue state transitions was to evaluate how cell-to-cell variability within a cell population could increase its risk of transitioning to a disease state. Including extrinsic noise in our stochastic simulations would allow us to see if there was an increase or decrease in the number of cells transitioning to a disease state. By observing the number of cells transitioning to these disease states, we could determine whether the addition of extrinsic noise is accompanied with a higher or lower risk of the tissue transitioning to a disease state. Indeed, we found that intrinsic noise could still allow for increased risk of transitions from the homeostatic state to the degenerative and tumorigenic states, while the inclusion of both extrinsic and intrinsic noise in the system further increased the risks for those transitions.

Here we did not consider the regulations of cell population at the tissue level, which includes cell proliferation, death, and various cell-cell communications between multiple cell types in the tissue. Future works can further expand the model by including these regulations to understand how the cell population size is maintained at a fixed tissue size, the transition to the degeneration state with smaller tissue size, or the transition to the tumorigenesis state with oversized tissue. For example, multiple tissue outcomes were found by modeling the Epithelial-Mesenchymal Communication (EMC) between tubular epithelial cells and interstitial fibroblasts in the renal system in response to acute tissue injuries [33]. The model can also be further expanded by including the spatial organization of the tissue. Agent-Based Model (ABM) modeling would be employed by considering the spatial distribution of the Hippo/YAP/TAZ pathway and cell-cell contact inhibition and communication [34]. It would be interesting to model the cross-talks between Hippo pathway and other signaling pathways via cell-autonomous or non-cell-autonomous approaches, such as WNT, SHH, TGF-b, JAK-STAT, EGFR, and Notch pathways, constitute a paracrine and/or autocrine signaling network [1]. Combining additional positive feedback loops with the proposed circuit may create other attractors that describe these other functions of the Hippo-YAP/TAZ pathway. It would also be interesting to see how more attractors emerge with these additional feedback loops by using the comprehensive network analysis introduced by Ye et al [19]. The potential roles of YAP/TAZ pathway on cancer metastasis would also be interesting to be studied with systems mathematical modeling. It is found that EMT promotes the activation of Hippo downstream nuclear effector YAP1 and TAZ [35], and ZEB1 can activate YAP1 by inhibiting its repressor miR-375 in prostate cancer [1], while YAP/TAZ activation can also induce EMT [36–38], either by controlling Smad2/3/4 nuclear accumulation and regulating downstream targets Snail, Slug, and Twist1 expression [39] or by inducing expression of ZEB1/2 [40, 41]. Study of the mutual regulation of YAP/TAZ and EMT regulatory network will helps us to understand the role of Hippo signaling pathway on cancer cell plasticity and metastasis.

## Materials and methods

### Nullclines and bifurcation analysis

To perform the nullclines analysis in Figs 2B and 2C and 3A and 3E, we used the reduced 2-ODE system. The [L]-nullcline and [*YT*_*up*_]-nullcline are determined by solving *d*[*L*]/*dt* = 0 and *d*[*YT*_*up*_]/*dt* = 0, respectively. The vector fields were calculated by first evaluating the 2-ODE system at the positions of the arrows. A custom function takes in these results and creates a grid of vectors. These vectors are all the same size but different colors based on their strength, which is calculated by using the magnitude of the vector. The steady states are found at the intersection points of two nullclines, and the stability of these steady states was determined by the signs of the real and imaginary parts of eigenvalues using software XPPAUT (http://www.math.pitt.edu/bard/xpp/xpp.html). The stability of these steady states was also confirmed using the 1-parameter bifurcation diagram in Figs 2A and 3C. Since we used a specific value of *k*_*YTup*0_, by checking the value of [*YT*_*up*_] in the Figs 2B and 2C, 3A and 2E, we can derive from which branch in the bifurcation diagram each steady state belongs to and determine its stability. The bifurcation analysis was performed using software Oscill8 (http://oscill8.sourceforge.net/).

### Sensitivity analysis

For our sensitivity analysis, to ensure the varied parameters are still within the biologically relevant range, we chose a value of 15% change to vary all the parameters individually, like our previous work [18]. Here, we define the fold change by the change in the saddle node’s position in terms of the *k*_*YTup*0_ value. To calculate the fold change in Fig 4A and 4B, we divided the x-coordinate of the saddle node’s position after a single parameter had been varied by the original x-coordinate of the saddle-node point (Fig 2A). We followed this procedure each time we either increased or decreased each of all the other parameters.

### Stochastic simulations

To determine the effect of intrinsic and extrinsic noise, we converted the 5-ODE system to a stochastic model, which is not a fully mass-action model as it still retains the Michaelis-Menten and Hill terms (Table D in S1 Text) [18, 42]. We used the Tau-leap-based Gillespie algorithm [26] to simulate the stochastic version of the model to include the intrinsic noise, where the system size factor Ω value was set to 100. To include extrinsic noise in the system, we varied all parameter values of the standard parameter set by 5% using Latin Hypercubic sampling. All simulations were ran for 720 time units to ensure the system reached a steady state.

## Supporting information

S1 Text **Fig A**. **Tristability due to Parameter *J*_*N*_ Variation**. One-parameter bifurcation diagram displays irreversibility from tumorigenic state to other states due to a decrease of 15% in the parameter *J*_*N*_. Possible state transitions are displayed with grey dashed line arrows. SN: Saddle node. **Fig B. Time courses for stochastic simulations display state transitions**. The dynamics of *YT*_*up*_, S, and N for 10 stochastic simulations when *k*_*YTup*0_ value was increased or decreased from the standard value *k*_*YTup*0_ = 0.007, which corresponds to the homeostatic state. **Table A. Description of differential equation terms**. **Table B. Variables of model**. **Table C. Parameters of differential equation system**. **Table D. Stochastic version of model**.
(PDF)
