# Supplementary material for: Control of tissue homeostasis, tumorigenesis, and degeneration by coupled bidirectional bistable switches
Source: PLoS Comput Biol. 2021 Nov 19;17(11):e1009606. doi: 10.1371/journal.pcbi.1009606 (PMC8641876; doi:10.1371/journal.pcbi.1009606)
Supplement: S1 Text — Fig A. Tristability due to Parameter JN Variation. One-parameter bifurcation diagram displays irreversibility from tumorigenic state to other states due to a decrease of 15% in the parameter JN. Possible state transitions are displayed with grey dashed line arrows. SN: Saddle node. Fig B. Time courses for stochastic simulations display state transitions. The dynamics of YTup, S, and N for 10 stochastic simulations when kYTup0 value was increased or decreased from the standard value kYTup0 = 0.007, which corresponds to the homeostatic state. Table A. Description of differential equation terms. Table B. Variables of model. Table C. Parameters of differential equation system. Table D. Stochastic version of model. (PDF) [file pcbi.1009606.s001.pdf]

S1 Supporting information

Control of Tissue Homeostasis, Tumorigenesis, and Degeneration by Coupled Bidirectional Bistable Switches

Diego Barra Avila[1], Juan R. Melendez-Alvarez[1], Xiao-Jun Tian[1,*]

[1]School of Biological and Health Systems Engineering, Arizona State University, Tempe, Arizona, United States of America.

* xiaojun.tian@asu.edu

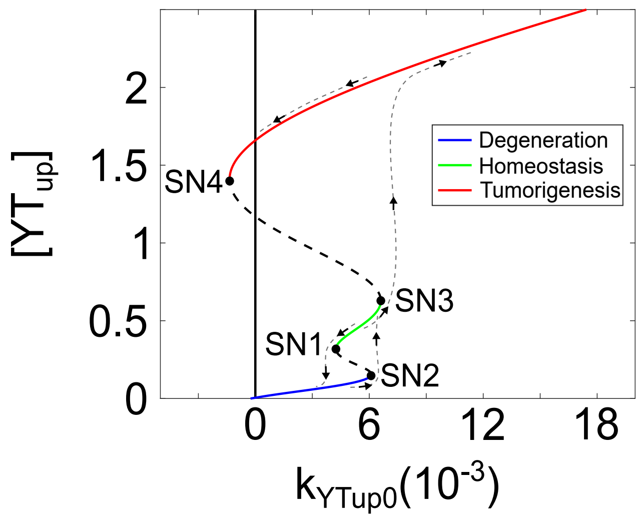

Fig A: **Tristability due to Parameter J_N Variation.** One-parameter bifurcation diagram displays irreversibility from tumorigenic state to other states due to a decrease of 15% in the parameter J_N. Possible state transitions are displayed with grey dashed line arrows. SN: Saddle node.

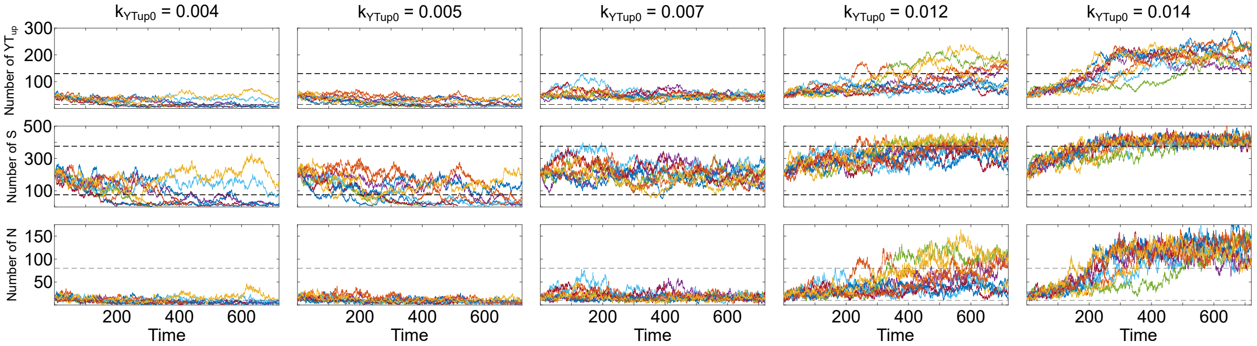

Fig B: **Time courses for stochastic simulations display state transitions.** The dynamics of YT_{up}, S, and N for 10 stochastic simulations when k_{YTup0} value was increased or decreased from the standard value $k_{YTup0} = 0.007$, which corresponds to the homeostatic state.

Table A: Description of differential equation terms

**Term**	**Description**
k_{L1}	Basal production of LATS1/2
$k_{L2} \cdot \frac{[YT_{up}]^n}{[YT_{up}]^n + J_L^n}$	Production of LATS1/2 induced by YAP/TAZ
$k_{L3} \cdot [L]$	Degradation of LATS1/2
k_{YTup0}	Basal production of YAP/TAZ
$k_{YTup1} \cdot \frac{[S]^n}{[S]^n + J_{YTup1}^n}$	Production of YAP/TAZ induced by SIRT1
$k_{YTup2} \cdot \frac{[N]^n}{[N]^n + J_{YTup2}^n}$	Production of YAP/TAZ induced by NOTCH
$k_{YTup3} \cdot \frac{[YT_{up}] \cdot [L]}{[YT_{up}] + J_{YTup3}}$	Phosphorylation of YAP/TAZ by LATS1/2
$k_{YTup4} \cdot \frac{[YT_p]}{[YT_p] + J_{YTup4}}$	Dephosphorylation of YAP/TAZ by other proteins
$k_{YTup5} \cdot [YT_{up}]$	Degradation of unphosphorylated YAP/TAZ
$k_{YTp1} \cdot [YTp]$	Degradation of phosphorylated YAP/TAZ
k_{S1}	Basal production of SIRT1
$k_{S2} \cdot \frac{[YT_{up}]^n}{[YT_{up}]^n + J_S^n}$	Production of SIRT1 induced by YAP/TAZ
$k_{S3} \cdot [S]$	Degradation of SIRT1
k_{N1}	Basal production of NOTCH
$k_{N2} \cdot \frac{[YT_{up}]^n}{[YT_{up}]^n + J_N^n}$	Production of NOTCH induced by YAP/TAZ
$k_{N3} \cdot [N]$	Degradation of NOTCH

Table B: Variables of model

**Variables**	**Description**	**Initial Values**
$[YT_{up}]$	Concentration of unphosphorylated YAP/TAZ	0.4732
$[YT_p]$	Concentration of phosphorylated YAP/TAZ	0.2387
$[L]$	Concentration of LATS1/2	1.6057
$[S]$	Concentration of SIRT1	2.0886
$[N]$	Concentration of NOTCH	0.1697

Table C: Parameters of differential equation system

Parameter	Description	Value	Units
k_{L1}	Basal production rate of LATS1/2	0.15	$\frac{concentration}{time}$
k_{L2}	Production rate of LATS1/2 induced by YAP/TAZ	0.2	$\frac{concentration}{time}$
J_L	Michaelis constant of YAP/TAZ-dependent activation of LATS1/2 production	0.05	$concentration$
k_{L3}	Degradation rate of LATS1/2	0.2166	$\frac{1}{time}$
k_{YTup0}	Basal production rate of YAP/TAZ	0.0073	$\frac{concentration}{time}$
k_{YTup1}	Production rate of YAP/TAZ induced by SIRT1	0.0225	$\frac{concentration}{time}$
J_{YTup1}	Michaelis constant of SIRT1-dependent activation of YAP/TAZ production	1	$concentration$
k_{YTup2}	Production rate of YAP/TAZ induced by NOTCH	0.105	$\frac{concentration}{time}$
J_{YTup2}	Michaelis constant of NOTCH-dependent activation of YAP/TAZ production	1	$concentration$
k_{YTup3}	Production rate of YAP/TAZ induced by LATS1/2	0.045	$\frac{1}{time}$
J_{YTup3}	Michaelis constant of LATS1/2-dependent activation of YAP/TAZ phosphorylation	1	$concentration$
k_{YTup4}	Rate of YAP/TAZ dephosphorylation	0.05	$\frac{concentration}{time}$
J_{YTup4}	Michaelis constant of protein-dependent activation of YAP/TAZ dephosphorylation	1	$concentration$
k_{YTup5}	Degradation rate of unphosphorylated YAP/TAZ	0.033	$\frac{1}{time}$
k_{YTp1}	Degradation rate of phosphorylated YAP/TAZ	0.05	$\frac{1}{time}$
k_{S1}	Basal production rate of SIRT1	0.01	$\frac{concentration}{time}$
k_{S2}	Production rate of SIRT1	1	$\frac{concentration}{time}$
J_S	Michaelis constant of YAP/TAZ-dependent activation of SIRT1 production	0.5	$concentration$
k_{S3}	Degradation rate of SIRT1	0.231	$\frac{1}{time}$
k_{N1}	Basal production rate of NOTCH	0.01	$\frac{concentration}{time}$
k_{N2}	Production rate of NOTCH	0.525	$\frac{concentration}{time}$
J_N	Michaelis constant of YAP/TAZ-dependent activation of NOTCH production	1.95	$concentration$
k_{N3}	Degradation rate of NOTCH	0.231	$\frac{1}{time}$
n	Hill coefficient	2	

The value for parameter k_{YTup0} in this table was used in the nullcline analysis in Fig 3 of the main text.

Table D: Stochastic version of model

**Term**	**Description**	**Propensity function**
$\emptyset \rightarrow L$	Basal production of LATS1/2	$k_{L1} \cdot \Omega$
$\emptyset \rightarrow L$	Induction of LATS1/2 by YAP/TAZ	$k_{L2} \cdot \frac{(\frac{YT_{up}}{\Omega})^n}{(\frac{YT_{up}}{\Omega})^n + J_L^n} \cdot \Omega$
$L \rightarrow \emptyset$	Degradation of LATS1/2	$k_{L3} \cdot L$
$\emptyset \rightarrow YT_{up}$	Basal production of YAP/TAZ	$k_{YTup0} \cdot \Omega$
$\emptyset \rightarrow YT_{up}$	Induction of YAP/TAZ production by SIRT1	$k_{YTup1} \cdot \frac{(\frac{S}{\Omega})^n}{(\frac{S}{\Omega})^n + J_{YTup1}^n} \cdot \Omega$
$\emptyset \rightarrow YT_{up}$	Induction of YAP/TAZ production by NOTCH	$k_{YTup2} \cdot \frac{(\frac{N}{\Omega})^n}{(\frac{N}{\Omega})^n + J_{YTup2}^n} \cdot \Omega$
$YT_{up} \rightarrow YT_p$	Phosphorylation of YAP/TAZ by LATS1/2	$k_{YTup3} \cdot \frac{(\frac{YT_{up} \cdot L}{\Omega^2})}{(\frac{YT_{up}}{\Omega}) + J_{YTup3}} \cdot \Omega$
$YT_p \rightarrow YT_{up}$	Dephosphorylation of YAP/TAZ	$k_{YTup4} \cdot \frac{(\frac{YT_p}{\Omega})}{(\frac{YT_p}{\Omega}) + J_{YTup4}} \cdot \Omega$
$YT_{up} \rightarrow \emptyset$	Degradation of unphosphorylated YAP/TAZ	$k_{YTup5} \cdot YT_{up}$
$YT_p \rightarrow \emptyset$	Degradation of phosphorylated YAP/TAZ	$k_{YTp1} \cdot YT_p$
$\emptyset \rightarrow S$	Basal production of SIRT1	$k_{S1} \cdot \Omega$
$\emptyset \rightarrow S$	Induction of SIRT1 production by YAP/TAZ	$k_{S2} \cdot \frac{(\frac{YT_{up}}{\Omega})^n}{(\frac{YT_{up}}{\Omega})^n + J_S^n} \cdot \Omega$
$S \rightarrow \emptyset$	Degradation of SIRT1	$k_{S3} \cdot S$
$\emptyset \rightarrow N$	Basal production of NOTCH	$k_{N1} \cdot \Omega$
$\emptyset \rightarrow N$	Induction of NOTCH production by YAP/TAZ	$k_{N2} \cdot \frac{(\frac{YT_{up}}{\Omega})^n}{(\frac{YT_{up}}{\Omega})^n + J_N^n} \cdot \Omega$
$N \rightarrow \emptyset$	Degradation of NOTCH	$k_{N3} \cdot N$

The system size parameter Ω has a unit of volume. Each of the species present within this table has a unit of molecular number rather than of *concentration*.
